# Vaccine resistant pseudorabies virus causes mink infection in China

**DOI:** 10.1186/s12917-018-1334-2

**Published:** 2018-01-19

**Authors:** Gui-sheng Wang, Yijun Du, Jia-qiang Wu, Fu-lin Tian, Xue-jie Yu, Jin-bao Wang

**Affiliations:** 1 Shandong Provincial Center for Animal Disease Control and Prevention, Jinan, China; 2 School of Life Sciences, Shandong University, 27 Shanda Road South, Jinan, 250100 China; 3 Shandong Key Laboratory of Animal Disease Control and Breeding, Institute of Animal Science and Veterinary Medicine, Shandong Academy of Agricultural Sciences, Jinan, China; 4 School of Health Science, Wuhan University, Wuhan, China

**Keywords:** Minks, Pseudorabies, Pseudorabies virus, Herpesvirus, China

## Abstract

**Background:**

Pseudorabies, a highly contagious infectious disease of swine is caused by pseudorabies virus (PRV). PRV can cause fatal infection in other animal species.

**Results:**

We report a deadly outbreak of pseudorabies that killed 87.2% (3522/4028) minks in a farm in 2014 in Shandong Province, China. PRV was isolated by using Vero cell culture and detected in mink samples by PCR from minks died during the outbreak. Epidemiological analysis indicated that 5.8% of minks (33/566) were PCR positive to PRV in Shandong Province. Phylogenetic analysis indicated that the PRV strains isolated from minks in this study were in the same clade with the Chinese porcine PRV isolates, which are resistant to the PRV vaccine.

**Conclusions:**

We demonstrated that pseudorabies virus caused an outbreak of minks in a farm in Shandong Province of China and the virus has a very high infection rate in minks in Shandong Province, which is a challenge for the fur industry in China.

## Background

Pseudorabies, also known as Aujeszky’s disease, is caused by pseudorabies virus (PRV), a herpesvirus of genus *Varicellovirus* in the family *Herpesviridae*. PRV causes diseases in domesticated and wild animals, but only swine is considered as the reservoir and animal host of PRV because swine are the only animal species that can survive an acute infection and have a latent infection [1, 2]. Like other alphaherpesviruses, in its natural host swine, PRV first invade epithelial cells of the mucosal surfaces, then the termini of nerve fibers, and finally the peripheral ganglia and the central nervous system to establish latent infection. In piglets or immunocompromised pigs, the nonspecific immune response is delayed or ineffective and the virus spreads through the central nervous system to cause abortion in pregnant swine and high mortality rates in piglets [2–4].

Due to its serious impact on the pig industry, some countries have tried to eradicate pseudorabies in commercial swine population based on the DIVA (differentiating infected from vaccinated individuals) vaccination program [5]. Pseudorabies has been eradicated in a few developed countries including the United States, several European countries and New Zealand [6–9]. Although the DIVA vaccination programs have been used in China, pseudorabies has reemerged since 2011 in China with increased infection frequency and severity in swine and other animals [10]. In recent years, a highly virulent PRV strain appeared in China that caused high mortality rates in grown pigs [4, 9]. The new PRV variant is resistant to vaccine immunization in vaccinated swine herds in many regions of China [10].

PRV can infect a broad range of domestic and wild animals with the exception of higher-order primates [11]. The hallmark of the PRV infection in non-porcine animal species is pruritus in the infected area and it is usually fatal PRV-infected mink exhibit neurological signs and die within a few days without pruritus [12].

PRV genome is a linear double-stranded DNA, approximately 150Kb with capacity to encode approximately 70 proteins [13]. PRV genome consists of a unique long region (UL) and a unique short region (US). The US region is bracketed by inverted repeat sequences, resulting in the formation of two possible PRV genome isomers with oppositely oriented US regions [13]. PRV major protective antigens including gB, gC, gD, and gH can stimulate animals to produce viral neutralization antibody and cellular immunity against the virus [14]. gE and TK genes are major PRV virulence genes. gE mediates cell fusion, virus diffusion between cells, and virion releasing and neurotropic tropism. It is also a marker gene to distinguish natural infection from vaccination since the vaccine lacks the gE gene. TK plays important roles in viral infections in the body and viral proliferation in nervous tissue. Although PRV infects several animal species, reported natural PRV infection of minks are rare [15, 16]. In September of 2014,3522 of 4028 minks died in 1 week in a farm in Shandong Province, China with 100% (3522/3522) case fatality and 87.2% (3522/4028) mortality. The clinical symptoms of the minks were sudden onset of pneumonia like syndroms, diarrhea and lethargy. Minks started to die 2 days after onset of illness and all of the dead minks died within a week. In this study, we studied the pathogen that caused the outbreak in minks in China.

## Methods

### Mink samples

**Fig. 1:**
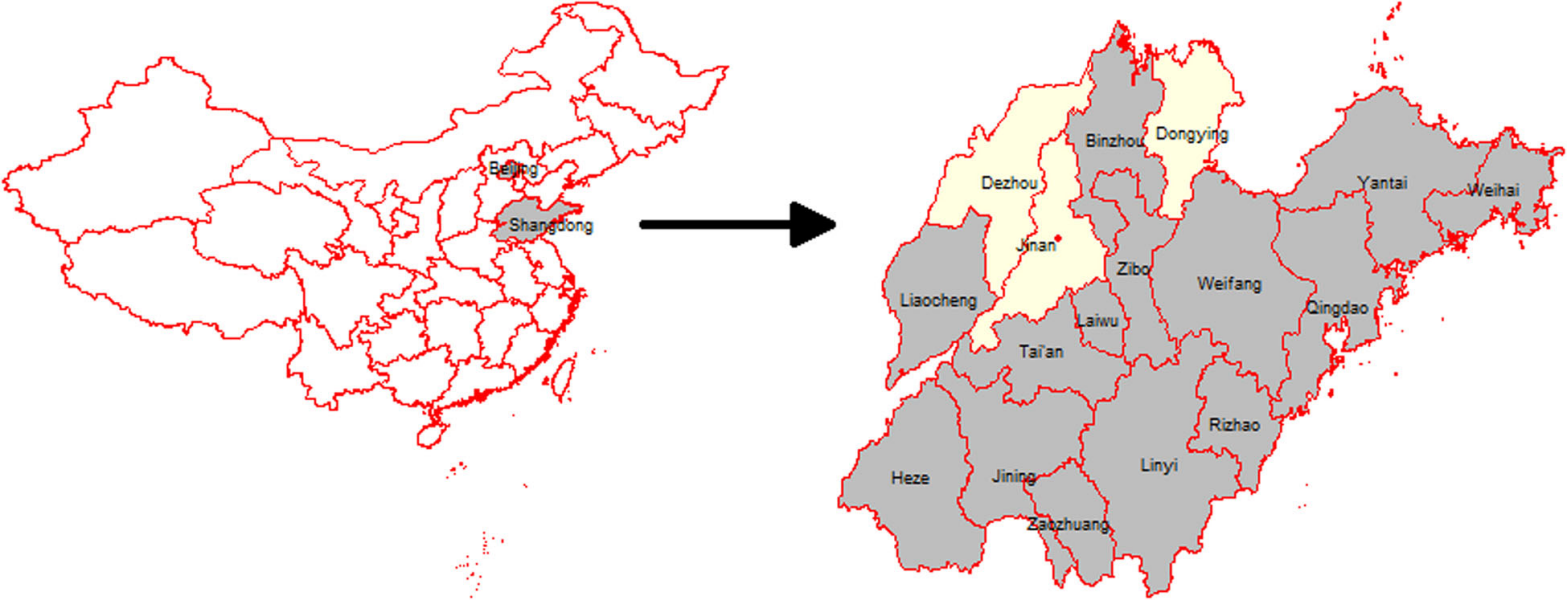
Geographic location of Shandong Province of China (left) and the mink sample collection sites (right). Mink samples were collected from 14 (grey areas) of 17 cities in Shandong Province. The maps were drawn using the R Project for Statistical Computing (https://www.r-project.org/)

We randomely seleted 10 dead minks during the outbreak from the farm to determine the causative agent. After the outbreak of mink infections on the intial farm, we obtained samples of 566 dead minks in 14 farms from 14 cities in Shandong Province to determine the ditribution and infection rate of the causative agent in minks in Shandong Province (Fig. 1). Sample size was determined according to a 95% confidence level and 10% PRV infection rate. The organs of the dead minks including heart, liver, spleen, lung, kidney, brain and intestine were collected from dead minks aseptically and were homogenized using metal beads (Tissue Lyser; Qiagen) in the RLT buffer (Qiagen).

### DNA and RNA extraction

Two hundred microliters of homogenized mink tissue supernatant from each organ was used for total nucleotide extraction with the MiniBEST Viral RNA/DNA Extraction Kit (Takara, Dalian, China) in accordance with the manufacturer’s protocol. The nucleic acid templates were stored at −80 °C until use.

### PCR and RT-PCR amplification and DNA sequencing

All the mink organs obtained above were tested for pseudorabies virus, influenza virus, parvovirus, rotavirus and porcine circovirus with PCR or RT-PCR. Porcine circovirus was amplified as previously described [17]. Pseudorabies virus, influenza virus, parvovirus, and rotavirus virus were amplified by using kit for each virus from Beijing Anheal Laboratories Co (Beijing, China) following the protocols of the manufacture.

For isolated strains of PRV, 6 genes including gB, gC, gD, gE, gH and TK were amplified by PCR (Table 1). High fidelity Taq enzyme AccuTaq™ LA DNA Polymerase (Sigma-Aldrich, Shanghai, China) was used for PCR in this study. For each reaction 1 μl of extracted DNA/RNA sample was used as template. The primers were designed in this study and the PCR cycles were the same for all primer pairs, which included a cycle of 95 °C for 3 min, 15 cycles of 95 °C for 30s, 65 °C for 30s (decreasing 1 °C each cycle) and 72 °C for 2 min, 15 cycles of 95 °C for 30s, 51 °C for 30s, and 72 °C for 2 min, and a final cycle of 72 °C for 10 min. The PCR products were electrophoresed on agarose gels and DNA bands with expected sizes were excised from the gels. The DNA purified from the gels was cloned into plasmid vector pMD18-T (Takara, Dalian, China). Three clones of each recombinant plasmid were sequenced on both stands by the Biotechnology Research Center, Shandong Academy of Agricultural Sciences (Jinan, China).

**Table 1:** Primers amplification of the entire CDS of gC, gD, gH, gE, and TK genes of PRV

Primers	Sequence	Position (bp)	Length (bp)	Annealing temperature	GenBank accession
gB-F	CTGGTGGCGGTCTTAGGCG	8–27	2738	60	KJ526439
gB-R	CTACAGGGCGTCGGGGTCC	2726–2744			
gC-F	GCTCGTGCAGGCGTACGT	53,326–53,343	1625	55	AF158090
gC-R	GCGGTCGTTTATTGATTCGG	54,950–54,931			
gD-F	CCCAGGTTCCCATACACTCAC	121,236–121,256	1258	55	AF086702
gD-R	TACTGCGGAGGCTACGGAC	122,487–126,469			
gE-F	AGACCATGCGGCCCTTTC	122,353–122,370	2710	58	AY249861
gE-R	ACGACCACTCCGTGTCCAGC	125,090–125,071			
gH-F	GGAGATGGGGGTGTGACC	59,631–59,648	3108	53	M61196
gH-R	GCGGCAGACACTTTAACTCTTG	62,701–62,680			
TK-F	AGGCGTTCGTAGAAGCGGT	59,195–59,213	1147	56	AY217095
TK-R	GGGCACGGCAAACTTTATTG	60,340–60,321			

### Phylogenetic analysis

In order to get comparable phylogenetic results for all genes, we only selected sequences for viruses that had complete genome sequences in GenBank to do phylogenetic analysis. The DNA sequences were aligned using the Clustal W program implemented in the MEGA7.0 software. The phylogenetic trees that reflected evolutionary distances were constructed using the Maximum-likelihood method employing a Kimura 2-parameter model for nucleotides reconstructed using the same software [18].

### Bacterial culture

The tissues of the brains and livers of minks were scratched with a bacterial inoculation loop and inoculated onto trypsin soy agar plates and blood agar plates. The plates were incubated at 37 °C for 48 h under aerobic and anaerobic conditions.

### Isolation of virus

The frozen brain and liver samples from the minks were homogenized, centrifuged at 7168 x g for 10 min at 4 °C, filtered through disposable filters with an average pore diameter of 0.22 μm and transferred to a Vero cell monolayer. The cells were cultured in DMEM supplemented with 2% fetal bovine serum at 37 °C and 5% CO_2_ condition for 72 h. The cultures were then harvested and repeatedly frozen and thawed three times. The suspension was used for passages on Vero cells. On the third passage of the cell cultures, the supernatant was tested with PCR using the PRV gB gene primers to determine infection with PRV.

## Results

### Bacterial pathogen detection

To determine whether the minks died of bacterial infection, their brain and liver tissue smears were stained by Gram staining and examed by microscopy. No bacterium was observered in these tissues from the Gram stain. Additionally, brain and liver samples of each mink were cultured on tryptic soy agar plates and blood agar plates. No bacterium was obtained from brain and liver tissues, suggesting that the minks were not infected with a bacterial pathogen.

### PRV as the causative agent of the mink outbreak

PCR amplification of PRV gE gene indicated that 100% (10/10) minks from the farm were positive to PRV with all organs tested incluing heart, liver, spleen, lung, kidney, brain and intestine. However, none of the organs was positive to other viruses including influenza virus, Parvovirus, rotavirus and porcine circovirus by PCR or RT-PCR amplification.

### Prevalence of minks to PRV

After demonstrating that the dead minks from the farm were infected with PRV, we further collected samples of 566 dead minks in 14 mink farms according to the 95% confidence level and 10% PRV infection rate. PCR amplification of gE gene showed that 5.8% (33/566) of minks were positive to PRV.

### Isolation of PRV from minks

To further determine the characteristics of PRV in the minks, we isolated PRV from mink tissues. On the third passage of the mink sample innoculated cell cultures, the supernatant of the culture was amplifed with PCR by using the gB gene primers. PCR amplification indicted that cells derived from two mink samples were positive. The viruses were named W-MPRV1 and W-MPRV2, respectively. Strain W-MPRV1 was isolated from the sample of a dead mink from the farm with outbreak of pseudorabies and W-MPRV2 was isolated from a dead mink from another farm during the epidemilogic investigation.

### Mink PRV sequence analysis

**Fig. 2:**
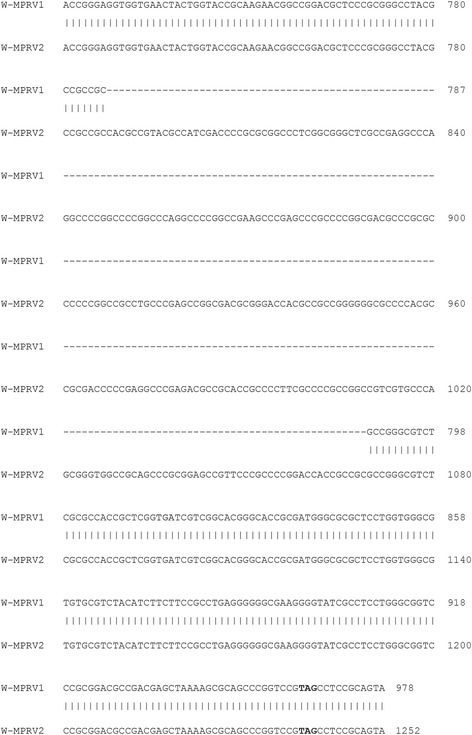
DNA sequence alignment of gD gene of mink isolates of PRV W-MPRV1 and W-MPRV2. W-MPRV1 had a deletion of 281 nucleotides from 787 nucleotide to 1069 nucleotide

**Fig. 3:**
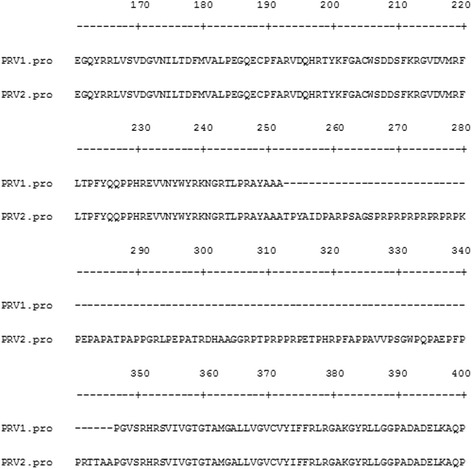
Amino acid sequence alignment of gD of mink isolates of PRV W-MPRV1 and W-MPRV2. W-MPRV1 had a deletion of 93 amino acids near the C-terminal

The entire coding region plus the 5′ and 3′ end of non-coding regions of gB (2735 bp), gC (1624 bp), gD (787 bp), gE (2724 bp), gH (3262 bp) and TK (1157 bp) of the PRV strains W-MPRV1 and W-MPRV2 were sequenced based on the PCR products of each gene. The DNA sequences of two genes (gB and TK) were 100% identical between the two mink PRV strains. The DNA sequence homology of three genes between the two mink PRV strains (gC, gE, and gH) were 99.8% to 99.9%. BLAST analysis indicated that homology of the strains W-MPRV1, W-MPRV2 and Chinese PRV isolates DL14/08(GenBank accession: KU360259), HNX (KM189912), HNB(KM189914), HN1201 (KP722022), HeN1(KP098534), TJ (KJ789182) were 99.9% to 100% for the 6 genes. The gD gene of strain W-MPRV1 was identical to strain W-MPRV2 and the Chinese PRV isolates on the 5′ end, but missed a 281 bp near the 3′ end of the gene compared to strain W-MPRV2 and the Chinese PRV isolates in GenBank (Fig. 2). The deletion of 281 nucleotides in the gD gene of W-MPRV1 caused a frameshift mutation and generated a protein with a deletion of 93 amino acids near the C-terminal (Fig. 3). The sequences generated in this study were deposited in GenBank (Accession numbers: MF940933 – MF940944).

### Phylogenetic analysis

**Fig. 4:**
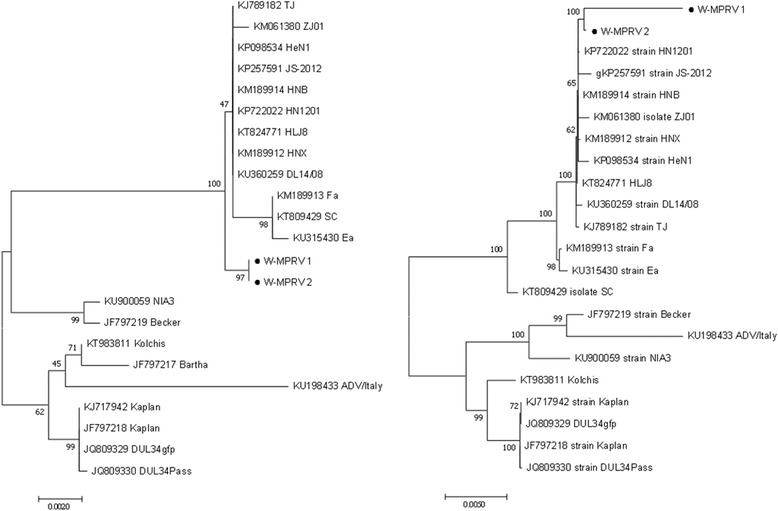
Phylogenetic tree of Pseudorabies virus. The phylogenetic tree was constructed with gB gene sequence (left) and the concatenated sequence of gB, gC, gD, gE, gH and TK genes (right) using MEGA5 software with 1000 replicates for bootstrap testing. Numbers (> 50) above or below branches are posterior node probabilities. The GenBank number was labeled on each line. Dots indicated sequences obtained in this study. Scale bar indicates nucleotide substitutions per site

Phylogenetic analysis with each individual gene and the concatenated sequence of gB, gC, gD, gE and TK genes showed similar results and only the phylogenetic trees of gB gene and the concatenated sequence were shown in Fig. 4. The PRV strains were classified into two branches with all 6 genes or the concatenated sequences with the Chinese isolates in one branch represented by strains W-MPRV1 and W-MPRV2 and the American and European isolates in another branch represented by Becker strain (Fig. 4).

## Discussion

In this study we investigated the causative agent of an outbreak of a fatal acute infectious disease in minks in a farm in Shandong Province of China, which caused a large scale of mink death with 100% cases fatality (3522 /3250) and 87.2% (3522/4028) mortality. We first screened the minks by PCR for pseudorabies virus, influenza virus, Parvovirus, rotavirus and porcine circovirus. Except for pseudorabies virus, all other viruses were negative in the mink samples. Our results indicated that PRV caused the outbreak of minks in the farm. To investigate whether PRV infection is widely distributed in Shandong Province, we subsequently collected samples of 566 minks from 14 farms in 14 cities of Shandong Province and amplified the gE gene with PCR. We found that 5.8% of 566 dead minks from all over Shandong Province were PCR positive to PRV. Our results further suggest that PRV infection of minks is widely spread in mink populations in Shandong Province.

We isolated two strains of pseudorabies virus from two dead minks from the farms. W-MPRV1 strain was isolated from a mink from the farm with pseudorabies outbreak and W-MPRV2 strain was isolated from a mink obtained in the subsequent epidemiologic investigation from another farm. Strains W-MPRV1 and W-MPRV2 were highly homologous, but not identical based on DNA sequences. They have identical sequences on gB and TK genes and 99.8% to 99.9% homology on gC, gE, and gH gene. The major difference between these two strains is that W-MPRV1 has a 281 bp deletion near the 3 end of the gD gene that generates a frameshift in gD gene resulting in a protein missing 93 amino acid near the C-terminal. Apparently the mutation in gD did not affect the virulence of W-MPRV1 to minks because it was isolated from a mink died in the outbreak. The gD of PRV is the viral ligand required for entry of extracellular particles [19, 20]. We do not know if the mutation generated a functional protein or not.

PRV strains from minks in this study are in the same phylogenetic clade with porcine PRV strains from China. This indicated that the mink PRV is a strain of porcine PRV. Among pigs, PRV is transmitted by contaminated secretions, excretions, aerosols, and venereal route [21]. No pig was present in the mink farm where PRV was isolated in this study and the farm was not near any pig farms. It is most likely the minks acquired PRV from meat products. A recent report indicated that farm raised foxes and domesticated dogs were infected with PRV transmitted by pork offal in China [22, 23]. A less likely possibility is that minks acquired PRV through long distance transmissions from aerosols because PRV can be transmit via the air [24]. In recent years porcine PRV infection has been a problem for pig farms in China. Bartha-K61 vaccine has been used worldwide to eradicate PRV [25]. Bartha-K61 vaccination provided complete protection against infection by the classic PRV SC strain. However, since late 2011 several highly virulent PRV strains including strains HeN1, JS-2012, and TJ have been isolated in various parts of China and the disease caused by these PRV strains is characterized by neurologic symptoms and a high mortality rate in pigs [25–27]. In protection assays, Bartha-K61 vaccine provided only partial protection against infection by strains HeN1, JS-2012, and TJ, suggesting that Bartha-K61 vaccine does not provide effective protection against new PRV strain infections in China [25–27]. The PRV strain isolate from minks in this study is phylogenetically closely related to the Bartha-K61 vaccine-resistant PRV strains, suggesting that vaccine-resistant PRV has spread widely throughout China and can cause a variety of animal infections. Eradicating pseudorabies in China under these circumstances proves challenging.

Shandong Province has the largest fur industry in China, which annually produces more than 50% of fur animals in China. In 2011, more than 30 million minks were raised in Shandong Province, with an annual increase of 20–40% [28]. This study indicated that PRV is widely distributed and can cause deadly outbreaks in minks in Shandong Province. Therefore, PRV infection of minks should be monitored and controlled in Shandong Province. Minks are primary fed fish and chicken products in Shandong Province. The source of PRV infected minks in Shandong Province is not clear. We also do not know whether PRV can establish persistent or prolonged infection in minks.

## Conclusions

We suggested that pseudorabies virus could cause an outbreak of minks in a farm in Shandong Province of China and the virus has a very high infection rate in minks in Shandong Province, which is a challenge for the fur industry in China.

## Abbreviation

PRV: Pseudorabies virus
